# Dihydrotestosterone treatment rescues the decline in protein synthesis as a result of sarcopenia in isolated mouse skeletal muscle fibres

**DOI:** 10.1002/jcsm.12122

**Published:** 2016-04-25

**Authors:** Oskar Wendowski, Zoe Redshaw, Gabriel Mutungi

**Affiliations:** ^1^ Department of Medicine, Norwich Medical SchoolUniversity of East AngliaNorwichNR4 7TJUK; ^2^ Faculty of Health and Life SciencesDe Montfort UniversityLeicesterUK

**Keywords:** Sarcopenia, Ageing, Skeletal muscle, Protein synthesis, Amino acid transporters

## Abstract

**Background:**

Sarcopenia, the progressive decline in skeletal muscle mass and function with age, is a debilitating condition. It leads to inactivity, falls, and loss of independence. Despite this, its cause(s) and the underlying mechanism(s) are still poorly understood.

**Methods:**

In this study, small skeletal muscle fibre bundles isolated from the extensor digitorum longus (a fast‐twitch muscle) and the soleus (a slow‐twitch muscle) of adult mice of different ages (range 100–900 days old) were used to investigate the effects of ageing and dihydrotestosterone (DHT) treatment on protein synthesis as well as the expression and function of two amino acid transporters; the sodium‐coupled neutral amino acid transporter (SNAT) 2, and the sodium‐independent L‐type amino‐acid transporter (LAT) 2.

**Results:**

At all ages investigated, protein synthesis was always higher in the slow‐twitch than in the fast‐twitch muscle fibres and decreased with age in both fibre types. However, the decline was greater in the fast‐twitch than in the slow‐twitch fibres and was accompanied by a reduction in the expression of SNAT2 and LAT2 at the protein level. Again, the decrease in the expression of the amino acid transporters was greater in the fast‐twitch than in the slow‐twitch fibres. In contrast, ageing had no effect on SNAT2 and LAT2 expressions at the mRNA level. Treating the muscle fibre bundles with physiological concentrations (~2 nM) of DHT for 1 h completely reversed the effects of ageing on protein synthesis and the expression of SNAT2 and LAT2 protein in both fibre types.

**Conclusion:**

From the observations that ageing is accompanied by a reduction in protein synthesis and transporter expression and that these effects are reversed by DHT treatment, we conclude that sarcopenia arises from an age‐dependent reduction in protein synthesis caused, in part, by the lack of or by the low bioavailability of the male sex steroid, DHT.

## Introduction

Sarcopenia, a skeletal muscle wasting as a result of old age, is a debilitating condition that is characterized by the progressive and gradual (rate 1–5% per annum depending on age) decline in skeletal muscle mass, strength, and function.^1^ It manifests itself predominantly in the elderly (E; generally in people >80 years) and leads to inactivity, increased susceptibility to falls, and eventually to loss of independence.^2^ Falls are a major health concern because they lead to injury, pain, hospitalization, and increased mortality. Despite its huge socioeconomic and health implications, the cause(s) and mechanism(s) underlying sarcopenia are still poorly understood.

In young (Y) adult men, skeletal muscle forms ~40–50% of body mass and acts as the main store of proteins.^3^ Therefore, in addition to its main physiological functions of respiration, postural support, and the control of movement, skeletal muscle is also important in the maintenance of body shape, structure, and composition. Skeletal muscle mass is maintained by the fine balance between protein synthesis and breakdown. Therefore, a decrease in skeletal muscle mass, such as that seen during ageing, must reflect an imbalance between these two processes. Although several studies have previously reported a 30–40% reduction in mixed protein synthesis with age,^4^, ^5^ the anabolic effects of amino acids are still intact in the E.^6^ Together, these findings suggest that sarcopenia may arise from a failure in the transmembrane transport of amino acids into and out of skeletal muscle fibres.

Amino acids enter and leave cells through highly specialized proteins known as amino acid transporters that are located on the plasma/cell membrane (=sarcolemma in skeletal muscle) and that play an important role in protein turnover.^7^ Although numerous amino acid transporters are present in cells,^8^ only two, the sodium‐coupled neutral amino acid transporter (SNAT) 2 and the sodium‐independent L‐type amino acid transporter (LAT) 2, have been consistently found in skeletal muscle in significant quantities.^9^, ^10^ Moreover, these transporters work together to transport essential amino acids into muscle cells (=muscle fibres). Thus, SNAT2 transports small neutral amino acids such as glutamine into muscle fibres. These are then exchanged by LAT2 for the branched chain, essential amino acids required for protein synthesis.^11^, ^12^ However, little is known about the effects of ageing on the number and function of these amino acid transporters in mammalian skeletal muscle fibres.

Sex steroids are important regulators of body shape, structure and, composition. For example, treating adult female rats,^13^ hypogonadal men,^14^ and E men with low testosterone (T) concentrations^15^ with T or any of its many derivatives for several weeks increases lean body mass and decreases fat content, suggesting that sex steroids are essential for the maintenance of skeletal mass and may be important in the management of sarcopenia. Although the concentration of free T in plasma decreases with age in both men and women,^16^ and T supplementation has been shown to increase lean body mass, T is not the most potent sex steroid in the body.^17^ Instead, in target tissues such as the skin, prostate, and brain, it is irreversibly metabolized to dihydrotestosterone (DHT) by the enzyme 5α‐reductase.^18^ Additionally, recent studies suggest that DHT, but not T, may be the more potent anabolic–androgenic steroid in mammalian skeletal muscle fibres.^19^ However, it is unknown whether DHT can reverse/arrest the development of sarcopenia.

The primary aims of this study were to investigate (i) the effects of ageing and DHT treatment on protein synthesis in mouse fast‐twitch and slow‐twitch skeletal muscle fibre bundles and (ii) the effects of ageing and DHT treatment on the expression and function of SNAT 2 and LAT 2 in intact mouse fast‐twitch and slow‐twitch skeletal muscle fibre bundles.

## Methods

### Small skeletal muscle fibre bundles

All the experiments reported here were performed at room temperature (~20°C) using small skeletal muscle fibre bundles isolated from the extensor digitorium longus (EDL, a fast‐twitch muscle that is composed mostly of type 2 fibres) and the soleus (a slow‐twitch muscle that is composed predominantly of type 1 fibres) muscles of Y (~100 days old), middle‐aged (M; ~350 days old) and E (>700 days old) female C57BL/6 mice killed by cervical disarticulation as recommended by UK legislation. Additionally, all the experiments conformed to the University of East Anglia Animal Welfare and Ethical Review Board guidelines. During the experiments, the muscles and muscle fibre bundles were bathed in mammalian Ringer's solution continuously bubbled with 95% O_2_ and 5% CO_2_ (for details of the Ringer's solution refer to^20^).

### Reverse transcription polymerase chain reaction

Total RNA was extracted from the EDL and soleus muscles of Y (*n* = 6), M (*n* = 6), and E (*n* = 6) mice using TRIzol^®^ reagent following the manufacturer's instructions (Life Technologies, Paisley, UK). Briefly, the muscles isolated from each animal were weighed, snap frozen in liquid nitrogen, pulverized, and mixed with 100 μL ice‐cold TRIzol. The mixture was centrifuged at 4°C, and the supernatant was mixed with chloroform. The aqueous phase was separated, mixed with isopropanol, and left to incubate at room temperature for 10 min. It was centrifuged, and the precipitated RNA was cleaned using an RNeasy Mini Kit (Qiagen, Manchester, UK) and redissolved in RNAse‐free water. The quality and quantity of the RNA extracted were determined spectrophotometrically, and 1 µg was reverse transcribed using QuantiTect Reverse Transcription Kit (Qiagen, Manchester, UK) following the manufacturer's instructions. Finally, the expression of SNAT2, LAT2, and α‐actin in the samples was determined after 35 cycles of amplification at 55°C using the following primers:

SNAT2:

Forward: 5′ ATCGTGGTGATTTGCAAGAA 3′
Reverse: 5′ GTCTGCGGTGCTATTGAATG 3′

LAT2

Forward: 5′ GCCCTCACCTTCTCCAACTA 3′
Reverse: 5′ CCATGTGAGGAGCAACAAAC 3′

α‐Actin

Forward: 5′ GCCCATCTATGAGGGCTATG 3′
Reverse: 5′ AATCTCACGTTCAGCTGTGG 3′

All the experiments were performed using muscles from three mice, and each experiment was repeated at least twice.

### Effects of ageing on the expression of SNAT2 and LAT2

To determine the effects of ageing on the expression of SNAT2 and LAT2, small muscle fibre bundles isolated from the EDL and the soleus muscle of Y (*n* = 4), M (*n* = 4), and E (*n* = 4) female mice were used. Within 2 min of isolation, the bundles were snap frozen in liquid nitrogen and pulverized. Cytosolic and membrane proteins were extracted using NP40 lysis buffer as previously described in.^21^ Finally, the proteins were immunoblotted for the expression of SNAT2 and LAT2 as described in the succeeding texts.

### Effects of dihydrotestosterone treatment on the expression of SNAT2 and LAT2

In another experiment, the effects of treating fibre bundles isolated from mice of different ages with physiological concentrations (~2 nM) of DHT^22^ were investigated. The bundles were divided into two equal groups. Group 1 (controls; *n* = 18 fibre bundles) was treated with the Ringer's solution containing 107.6 μM ethanol (the vehicle used to dissolve the DHT) for 1 h. Group 2 (experiment; *n* = 18 fibre bundles) was treated for the same duration with Ringer's solution containing 630 pg ml^−1^ (2.17 nM) DHT (5α‐Androstan‐17β‐ol‐3‐one; Sigma, Gillingham, Dorset, UK). At the end of the treatments, the bundles were snap frozen in liquid nitrogen, pulverized, and proteins were extracted as described in.^21^ Finally, the proteins were immunoblotted for the expression of SNAT2 and LAT2 as described in the succeeding texts.

### Effects of ageing and DHT treatment on protein synthesis

The effects of ageing and DHT treatment on protein synthesis were determined as previously described in.^12^ Briefly, fast‐twitch and slow‐twitch muscle fibre bundles isolated from Y and E mice were divided into two equal groups. Half (controls; *n* = 12 fibre bundles) was treated for 1 h with the standard Ringer's solution containing 107.6 μM ethanol plus 2 mM C^14^‐labelled isoleucine [L‐(U‐^14^ C) Ile; radioactivity level 3.46 μCi ml^−1^] (purchased from PerkinElmer, Buckinghamshire, UK). The other half (treatment/experimental group; *n* = 12 fibre bundles) was treated with the Ringer's solution containing 2.17 nM DHT plus L‐[U‐^14^ C] Ile for 1 h. At the end of the experiment, the fibre bundles were washed with ice‐cold phosphate buffered saline and the excess phosphate buffered saline was blotted. The bundles were snap frozen in liquid nitrogen, pulverized, and mixed with NP40 buffer. The proteins were precipitated using trichloroacetic acid, washed three times with ice cold acetone, and redissolved in 200 μL of KOH. The amount of proteins in each lysate was determined using the quick Bradford assay, and the remaining lysate was mixed with an equal amount of Ecoscint A liquid scintillant (Fisher Scientific, Leicestershire, UK). The radioactivity of the mixture was determined 1 h and 24 h after the end of each experiment using a HIDEX 300SL liquid scintillation counter (Lablogic Systems Ltd, Sheffield, UK). Additionally, each experiment was repeated at least twice.

### Immunoblotting

To determine the effects of ageing and DHT treatment on the expression of SNAT2 and LAT2 proteins in the two muscle fibre types, equal amounts of the proteins collected from the experiments described in the preceding texts were immunoblotted as previously described in.^12^, ^21^ Briefly, 10 µg of the proteins was separated by standard gel electrophoresis and transferred onto nitrocellulose membranes. The membranes were blocked for non‐specific antibody binding using 5% milk. They were then immunoblotted for the expression of SNAT2 (sc‐166366; Santa Cruz Biotechnology, Santa Cruz, Ca, USA) and LAT2 (ab123893) from Abcam (Abcam, Cambridge, UK). The peEF2 antibody was a gift from Prof. Chris Proud, University of Southampton. Finally, they were visualized using SuperSignal WestPico chemiluminescence substrate (Perbio Science UK Ltd, Cramlington and Northumberland, UK) and exposure to film.

To check for loading, the membranes were stripped and reprobed with a pan‐actin antibody (ab3280, Abcam, Cambridge, UK). All the blots were run in duplicate, and each experiment was repeated at least twice.

To determine the expression of SNAT2 and LAT2 in the various muscle fibre bundles, the blots were digitized, and the intensity of the various mRNA and protein bands were analysed using Scion Image^®^ from NIH and normalized to that of the loading control (actin).

## Statistical analysis

Statistical analysis of the data was performed using SPSS statistical program (IBM, UK). The data obtained from each fibre type under control and after the various treatments were compared using a one‐way ANOVA with Bonferroni *post hoc* test and a *P* < 0.05 was considered statistically significant.

## Results

### Effects of ageing on skeletal muscle mass

**Figure 1:**
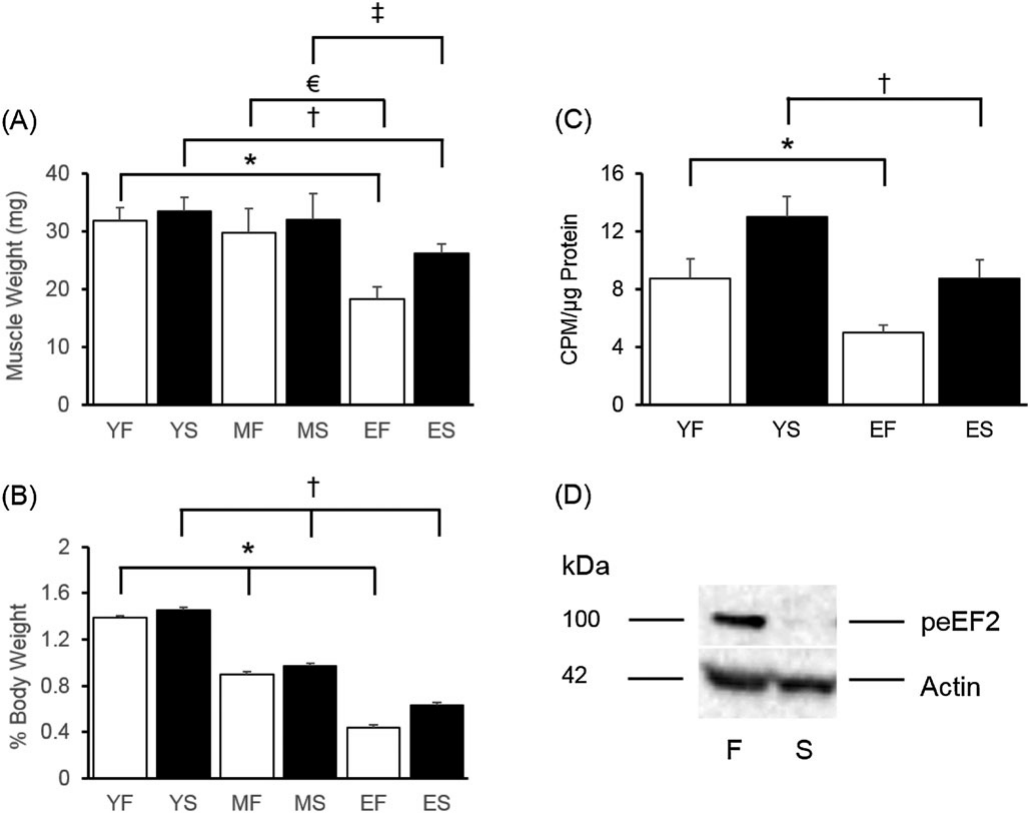
Ageing decreases skeletal muscle mass and protein synthesis. Bar graphs showing the effects of ageing on muscle weight (A and B) and protein synthesis (C) in the extensor digitorium longus (F; clear bars) and soleus (S; filled bars) of young (Y), middle‐aged (M) and elderly (E) mice. (D) A typical Western blot showing the phosphorylation of eEF2 in the extensor digitorium longus (F) and soleus (S) of mice ~100 days old. Note that ageing decreases skeletal muscle mass and protein synthesis in both fibre types. Additionally, the extensor digitorium longus expresses more phosphor‐eEF2 than the soleus. The data in (A) and (B) are from whole muscles, whereas those in (C) and (D) are from small muscle fibre bundles. *†*P* < 0.05 when the data from the elderly and the middle‐aged mice were compared with that from the young mice. € ± *P* < 0.05 when the data from the middle‐aged mice were compared with those from the elderly mice.

Figure 1 shows some of the basic observations from this study. As the results show, ageing led to a marked decline in the weight of both the EDL and soleus muscles (Figure 1A and B). For example, the EDL and soleus muscles from 100‐day‐old mice weighed 31.9 ± 2.2 mg and 33.5 ± 2.4 mg, respectively. However, by the time the mice were ~730 days old, the weight of the muscles had declined to 18.3 ± 2.1 mg and 26.3 ± 1.6 mg, respectively (Figure 1A). The effects became more apparent when the muscle weights were normalized to body mass (Figure 1B). Presented this way, the weight of the EDL decreased from ~1.4% body weight in the 100‐day‐old mice to 0.4% body weight in the 730‐day‐old mice (Figure 1B).

### Effects of ageing on protein synthesis

As the results displayed in Figure 1C show, at all ages investigated, protein synthesis was always higher in the slow‐twitch than in the corresponding fast‐twitch muscle fibres. Also, the difference in protein synthesis between the two fibre types increased with age. Thus, protein synthesis in the slow‐twitch fibres isolated from 100‐day‐old mice was ~49% higher than that of the fast‐twitch fibres from the same mice. However, by the time the mice were 730 days old, this difference had risen to ~75% (Figure 1C).

As the results also show, ageing led to a marked decrease in protein synthesis in both fibre types (Figure 1C). However, the effects were greater in the fast‐twitch fibres than in the slow‐twitch fibres. Thus, ageing led to a 42 ± 3.4% decline in protein synthesis in the fast‐twitch fibres but only to a 33 ± 2.7% decrease in the slow‐twitch fibres (Figure 1C).

The phosphorylation of eukaryotic elongation factor (eEF) 2 in fast‐twitch and slow‐twitch fibres is shown in Figure 1D. As the results show, the phosphorylation of eEF2 was always higher in the fast‐twitch than in the slow‐twitch muscle fibres (Figure 1D).

### Effects of ageing on the expression of SNAT2 and LAT2

**Figure 2:**
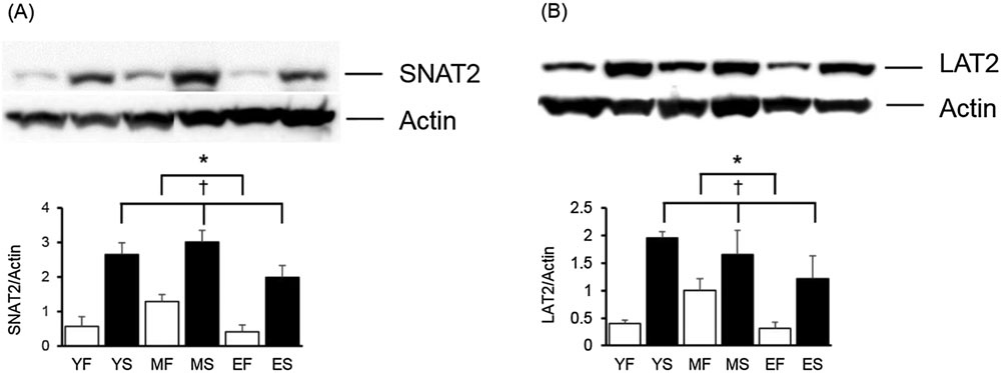
Ageing decreases the expression of sodium‐coupled neutral amino acid transporter 2 and LAT2 proteins. Bar graphs and typical immunoblots showing the effects of ageing on the expression of sodium‐coupled neutral amino acid transporter 2 and L‐type amino‐acid transporter proteins in small muscle fibre bundles isolated from the extensor digitorium longus (F; clear bars) and soleus (S; filled bars) of young (Y), middle‐aged (M), and elderly (E) mice. Note that ageing decreases the expression of both proteins in the fast‐twitch and slow‐twitch fibre bundles. *†*P* < 0.05 when the data from the elderly and the middle‐aged mice were compared with those from the young mice.

As mentioned in the ‘Introduction’ section, SNAT2 and LAT2 work together to transport the branched chain, essential amino acids that are required for protein synthesis into skeletal muscle fibres. Therefore, in another experiment, we determined whether the reduction in protein synthesis reported in the preceding texts was a result of an age‐dependent decline in the number of amino acid transporters. As the results displayed in Figure 2 show, ageing significantly decreased in the expression of SNAT2 and LAT2 proteins in both muscle fibre types. Moreover, the decrease in SNAT2 was greater than that of LAT2 in both fibres types. It was also higher in the fast‐twitch fibres (~35%) than in the slow‐twitch fibres (~25%).

**Figure 3:**
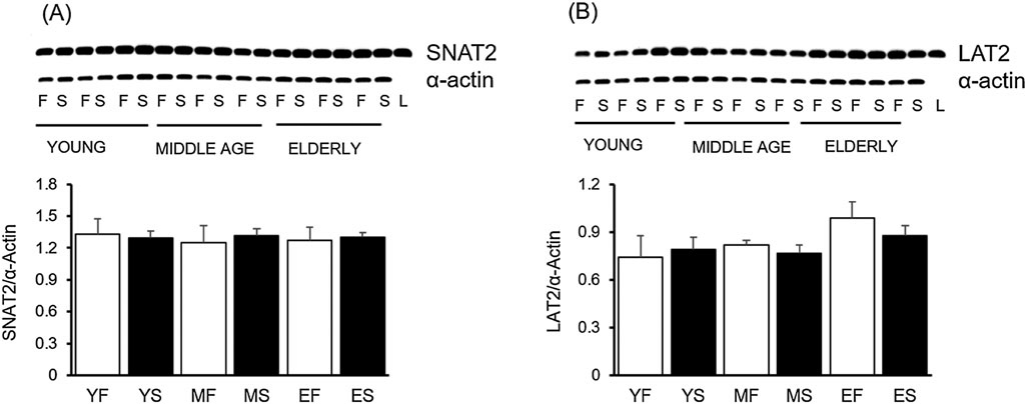
Ageing has no effect on sodium‐coupled neutral amino acid transporter 2 and L‐type amino‐acid transporter mRNA. Bar graphs and immunoblots showing the effects of ageing on the expression of sodium‐coupled neutral amino acid transporter 2 and L‐type amino‐acid transporter mRNA in small muscle fibre bundles isolated from the extensor digitorium longus (F; clear bars) and soleus (S; filled bars) of young (Y), middle‐aged (M), and elderly (E) mice. Note that ageing has no significant effect on the mRNA for both proteins in the fast‐twitch and slow‐twitch fibre bundles. L, liver samples.

To determine the mechanism underlying this age‐dependent decline in SNAT2 protein expression and function, we investigated the effects of ageing on SNAT2 and LAT2 mRNA. As the results displayed in Figure 3 show, ageing had no effect on the mRNA for both transporters in the two muscle fibre types (Figure 3A and B), suggesting that lack of mRNA was not the cause of the age‐dependent decline in SNAT2 and LAT2 expressions at the protein level.

### Effects of DHT treatment on protein synthesis and amino acid transporter expression

**Figure 4:**
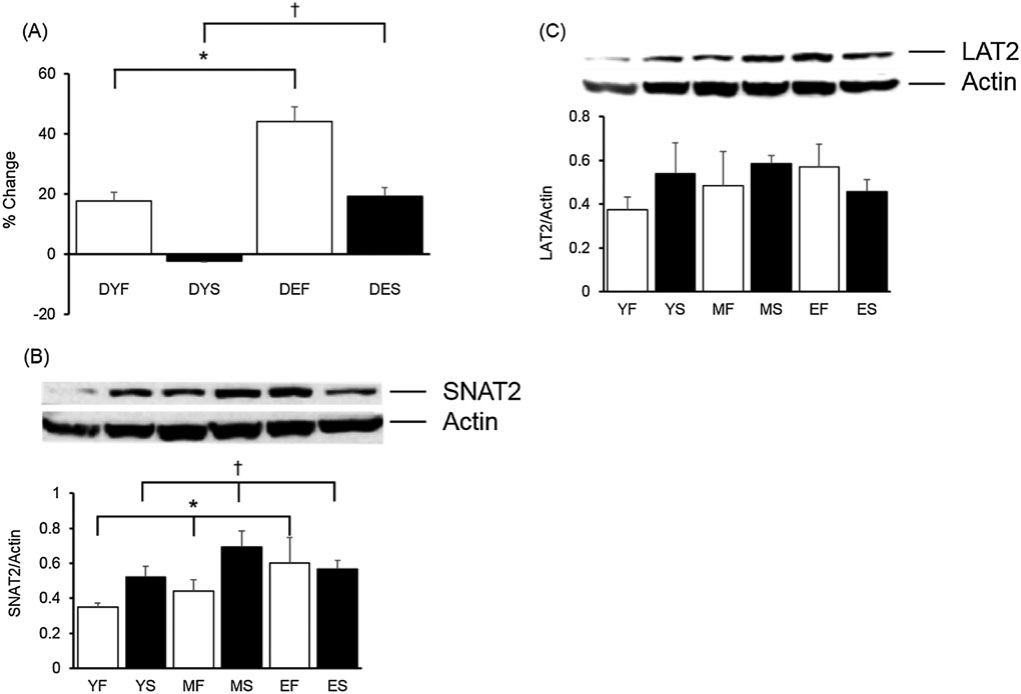
Dihydrotestosterone treatment reverses the effects of age on protein synthesis and the expression of sodium‐coupled neutral amino acid transporter 2 and L‐type amino‐acid transporter proteins. Bar graphs and typical western blots showing the effects of treating small muscle fibre bundles isolated from the extensor digitorium longus (F; clear bars) and soleus (S; filled bars) of young (Y), middle‐aged (M), and elderly (E) mice with physiological concentrations of dihydrotestosterone for 1 h on protein synthesis (A), sodium‐coupled neutral amino acid transporter 2 protein expression (B), and L‐type amino‐acid transporter protein expression (C). Note that treating the fibre bundles with dihydrotestosterone reverses the age‐dependent decline in protein synthesis as well as the age‐dependent decrease in sodium‐coupled neutral amino acid transporter 2 and L‐type amino‐acid transporter protein expression. *†*P* < 0.05 when the data from the elderly and the middle‐aged mice were compared with those from the young mice.

To determine whether the decrease in protein synthesis with ageing was a result of low concentrations of DHT in muscles, we investigated the effects of treating fast‐twitch and slow‐twitch skeletal muscle fibre bundles with physiological concentrations of DHT. As the results displayed in Figure 4A show, treating the fibre bundles isolated from Y mice with DHT had no effect on protein synthesis in the slow‐twitch muscle fibres. However, it led to a 17.8 ± 3% increase in protein synthesis in the fast‐twitch fibres.

In contrast, treating the fibre bundles isolated from E mice with DHT completely reversed the age‐dependent decline in protein synthesis. Thus, in the E mice, the DHT treatment led to a 44 ± 5% and a 21 ± 3% increase in protein synthesis in the fast‐twitch and slow‐twitch fibre bundles, respectively (Figure 4A).

DHT treatment also abolished the age‐dependent decline in SNAT2 (Figure 4B) and LAT2 (Figure 4C) protein in both muscle fibre types.

## Discussion

### Protein synthesis in fast‐ and slow‐twitch fibres

A key finding in the present study is the observation that, at all ages investigated, protein synthesis was always higher in the slow‐twitch (type 1) than in the fast‐twitch (type 2) fibres, and this difference became greater with age. Although type 1 fibres isolated from the vastus lateralis of human subjects have been shown to have higher fractional protein synthesis rates than type 2a fibres from the same muscle,^23^ this is the first study to show that the difference in the rate of protein synthesis between the two fibre types increases with age. In previous studies, the difference was attributed to the levels of phosphorylated eEF2 and adenosine monophosphate‐activated protein kinase present in the fibre types.^23^ In mammalian skeletal muscles, the phosphorylation of these proteins is higher in fast‐twitch than in slow‐twitch fibres and is associated with decreased protein synthesis. However, from the current and previous^12^ findings, we do not think that the phosphorylation of eEF2 and adenosine monophosphate‐activated protein kinase is the only cause of the fibre type differences in protein synthesis. Our results suggest that other factors such as the levels of amino acid transporters expressed by the various muscle fibres may also play a role.

### Effects of ageing on protein synthesis in mammalian skeletal muscle fibres

Another important finding in the present study was the observation that protein synthesis in both fibre types decreased with age and that the decline was greater in the fast‐twitch fibres than in the slow‐twitch fibres. Although a number of previous studies have reported a reduction in the fractional synthetic rate of mixed muscle and specific myofibrillar proteins with age,^3^, ^24^, ^25^ these studies were performed on muscle biopsies from human subjects. Moreover, most of the biopsies were from muscles such as the quadriceps femoris and vastus lateralis that consist of a mixture of fast‐twitch and slow‐twitch fibres and until recently, it was impossible to separate the fractional synthesis rate of the various fibre types.^23^ Therefore, from these studies, it was impossible to attribute the age‐dependent decline in protein synthesis to a specific fibre type. Although ageing has also been shown to decrease the fractional synthetic rates of type 2a and 2x myosin heavy chains in human muscle biopsies,^3^, ^24^ this is the first study to show that the effects of ageing on protein synthesis are greater in fast‐twitch than in slow‐twitch fibres, and that this difference increases with age. Together, these findings suggest that sarcopenia may arise, in part, from an age‐dependent decline in muscle protein synthesis.

### Effects of ageing on amino acid transporter expression in mammalian skeletal muscle fibres

In the present study, the decrease in protein synthesis with age was accompanied by a decline in both SNAT2 and LAT2 proteins. SNAT2 is a system A amino acid transporter that belongs to the SLC38 gene family. It mediates the sodium‐coupled transport of small neutral amino acids such as glutamine, alanine, etc. into muscle fibres. These are then exchanged, by LAT2, for the large branched chain amino acids such as leucine, histidine, etc. that are essential for protein synthesis in skeletal muscle.^12^ Like the decrease in protein synthesis with age, the decline in SNAT2 expression was greater in the fast‐twitch fibres than in the slow‐twitch fibres. Here, we speculate that the decrease in protein synthesis and the expression of SNAT2 with age are linked and that they are responsible for the development of sarcopenia.

Previously, one of the reasons advanced to explain the decline in protein synthesis with age was the reduction in the mRNA required for protein translation.^26^ For example, the age‐dependent decline in type 2a and 2x myosin heavy chains was associated with a large decrease in the mRNA for these proteins.^24^ However, as the results reported here show, ageing did not affect the SNAT2 and the LAT2 mRNA levels in mouse skeletal muscle fibres. Together, these findings suggest that the effects of age on mRNA expression may vary from protein to protein, and that the changes in the expression of SNAT2 and LAT2 may arise from post‐transcriptional control of mRNA translation. Indeed, a number of studies have previously reported changes in the expression of various microRNAs with age.^27^ However, the role microRNAs play in the age‐dependent decline in protein synthesis and hence in the development of sarcopenia remains uncertain.

### Effects of DHT treatment on the age‐dependent decline in protein synthesis and amino‐acid transporter expression

A novel finding in the present study was the observation that treating small skeletal muscle fibre bundles isolated from E mice with physiological concentrations of DHT rescues the age‐dependent decline in protein synthesis in both muscle fibre types. Moreover, the effects of DHT treatment, like those of ageing on protein synthesis, were greater in the fast‐twitch than in the slow‐twitch fibres. DHT treatment was also accompanied by an increase in the expression of SNAT2 and LAT2 in both fibre types.

In humans, from the age of 50 years onwards, the plasma levels of T decrease progressively with age, whereas ageing has the opposite effect on the plasma concentration of steroid hormone binding globulin whose concentration increases.^22^, ^28^ Both changes severely curtail the levels of free plasma T and hence its bioavailability. Moreover, the age‐dependent decrease in plasma T concentrations parallels the decline in skeletal muscle mass and function with age, suggesting that the lack of or the reduced bioavailability of T may be one of the causes of sarcopenia.

Although a number of studies have previously demonstrated the beneficial effects of T replacement therapy (TRT), recent studies suggest that DHT and not T may be the main anabolic hormone in skeletal muscle.^12^, ^19^ It has also been suggested that T may exert its anabolic effects in skeletal muscle through the intramuscular release of an intermediary such as insulin‐like growth factor 1.^29^ Here, we speculate that this intermediary is DHT.

As mentioned in the preceding texts, DHT treatment increases the expression of both SNAT2 and LAT2 protein in E muscle fibres. Although the mechanism involved was not investigated in the present study, the rapidity with which it occurred suggest that it involves the recruitment of SNAT2 and LAT2 heavy chains from an intracellular compartment. The expression of SNAT2 is regulated by many factors including pH, amino acid deprivation, and growth factors such as insulin and insulin‐like growth factor 1. Moreover, like in the current study, these factors increase the expression and function of SNAT2 within minutes.^30^ It is also thought that these effects do not involve transcription and translation. Instead, it is thought that they stimulate the recruitment of SNAT2 from an intracellular compartment in a similar manner to that of insulin on GLUT4 activation.^30^ However, whether DHT increases the expression of SNAT2 and LAT2 protein through a similar mechanism is uncertain.

### Use of DHT in the management of sarcopenia

As mentioned in the preceding texts, the free plasma concentration of T (i.e. bioavailable T) in men decreases with age, and the decline corresponds well with the onset and progression of sarcopenia.^22^, ^28^ This finding has fuelled many trials in the use of TRT as a possible treatment for sarcopenia. However, in E men, TRT reverses some but not all the effects of sarcopenia.^17^, ^22^ It is also associated with several side effects including an increase in the risk of developing prostate cancer and gynecomastia, alterations in the composition of plasma lipids, and increased cardiovascular events. Moreover, most of these adverse effects are mostly a result of the aromatization of T to oestrogen by the enzyme aromatase and its reduction to DHT by 5α‐reductase,^17^, ^31^ suggesting that the use of a non‐metabolizable anabolic–androgenic steroid may be a safer option.

One such steroid is DHT. However, very few studies have investigated the clinical use of DHT in the management of sarcopenia. DHT has many advantages over T. Firstly, it is neither aromatized nor reduced in target tissues. Secondly, in peripheral tissues, most of the DHT is thought to be produced locally,^22^ suggesting that its use can be targeted at specific tissues. Thirdly, treatment of E men with a transdermal DHT gel has been shown to be effective in the management of some of the effects of sarcopenia without the side effects of T.^32^, ^33^ Fourthly, the results we report here suggest that DHT treatment may be able to reverse the effects of ageing on protein synthesis and amino acid transporter expression. Together, these factors suggest that DHT may be a better therapeutic target for the management of sarcopenia than T.

### Summary

The results reported in this study show that at all ages investigated, protein synthesis was always higher in slow‐twitch (type 1) fibres than in fast‐twitch (type 2) fibres and that it decreased with ageing in both fibre types. Furthermore, the decline of protein synthesis with age was always greater in the fast‐twitch fibres than in the slow‐twitch fibres and was accompanied by a corresponding reduction in the levels of SNAT2 and LAT2 proteins but not their mRNA. The findings also show that treating the muscle fibre bundles with physiological concentrations of DHT abolished the effects of ageing on both protein synthesis and amino acid transporter expression, suggesting that sarcopenia may arise from an age‐dependent decline in protein synthesis caused, in part, by lack of or the low bioavailability of DHT. From these findings, we postulate that DHT treatment may have clinical implications in the management of sarcopenia.

## Conflict of interests

None declared.
